# Pharmacological Validation of Long-Term Treatment with Antiretroviral Drugs in a Model of SIV-Infected Non-Human Primates

**DOI:** 10.3390/pharmaceutics14112282

**Published:** 2022-10-25

**Authors:** Thibaut Gelé, Hélène Gouget, Nathalie Dereuddre-Bosquet, Valérie Furlan, Roger Le Grand, Olivier Lambotte, Delphine Desjardins, Aurélie Barrail-Tran

**Affiliations:** 1 Immunologie des Maladies Virales, Auto-Immunes, Hématologiques et Bactériennes, Université Paris-Saclay, Inserm, CEA, 92265 Fontenay-aux-Roses, France; 2 Service de Pharmacologie-Toxicologie, Hôpital Bicêtre, AP-HP. Université Paris-Saclay, 94275 Le Kremlin-Bicêtre, France; 3 Immunologie des Maladies Virales, Auto-Immunes, Hématologiques et Bactériennes, Service de Médecine Interne Immunologie Clinique, Hôpital Bicêtre, Université Paris-Saclay, AP-HP, Inserm, CEA, 94275 Le Kremlin-Bicêtre, France; 4 Immunologie des Maladies Virales, Auto-Immunes, Hématologiques et Bactériennes, Service de Pharmacie, Hôpital Bicêtre, Université Paris-Saclay, AP-HP, Inserm, CEA, 94275 Le Kremlin-Bicêtre, France

**Keywords:** tenofovir/emtricitabine/dolutegravir, pharmacokinetics, long-term treatment, non-human primate

## Abstract

The development of animal models undergoing long-term antiretroviral treatment (ART) makes it possible to understand a number of immunological, virological, and pharmacological issues, key factors in the management of HIV infection. We aimed to pharmacologically validate a non-human primate (NHP) model treated in the long term with antiretroviral drugs after infection with the pathogenic SIVmac251 strain. A single-dose pharmacokinetic study of tenofovir disoproxil fumarate, emtricitabine, and dolutegravir was first conducted on 13 non-infected macaques to compare three different routes of administration. Then, 12 simian immunodeficiency virus (SIV)-infected (SIV^+^) macaques were treated with the same regimen for two years. Drug monitoring, virological efficacy, and safety were evaluated throughout the study. For the single-dose pharmacokinetic study, 24-h post-dose plasma concentrations for all macaques were above or close to 90% inhibitory concentrations and consistent with human data. During the two-year follow-up, the pharmacological data were consistent with those observed in humans, with low inter- and intra-individual variability. Rapid and sustained virological efficacy was observed for all macaques, with a good safety profile. Overall, our SIV^+^ NHP model treated with the ART combination over a two-year period is suitable for investigating the question of pharmacological sanctuaries in HIV infection and exploring strategies for an HIV cure.

## 1. Introduction

In the management of people living with HIV (PLWHIV), current antiretroviral treatment (ART) is effective in achieving and maintaining undetectable plasma HIV-RNA levels [1]. However, it does not eradicate the virus from reservoirs [2]. Among the factors of viral persistence is the presence of long-lived cells harbouring replication-competent HIV in a latent state coupled with insufficient tissular diffusion of current antiretroviral drugs (ARVs), leading to subtherapeutic concentrations and the establishment of pharmacological sanctuaries [3,4,5,6]. A better understanding of the mechanisms of HIV persistence is crucial for the optimisation of strategies to achieve an HIV cure [7]. Studies to investigate determinants of HIV reservoirs, such as the distribution of antiretroviral drugs in tissues or immunological responses, require access to tissues and cells [8,9,10].

Animal models are critical for tissue-based studies. Non-human primates (NHPs) can be infected with the pathogenic simian immunodeficiency virus (SIV), which recapitulates many of the characteristics of HIV infection and AIDS in humans [11,12]. The use of this animal model over the last several decades has allowed a better understanding of the disease [13]. Comparisons of the results obtained in NHP models with those observed in human studies will lead to further improvement in our scientific knowledge [14,15,16], especially for the development of therapeutic strategies towards an HIV cure.

Access to NHP models that show plasma exposure similar to that in humans and robust suppression of viremia is a prerequisite for understanding the mechanisms of viral persistence under suppressive ART. We selected tenofovir (TNF) disoproxil fumarate (TDF), emtricitabine (FTC), and dolutegravir (DTG), as they are among the preferred first-line treatment options worldwide [17,18,19,20].

This study had two main objectives: (1) to estimate and compare plasma pharmacokinetic (PK) parameters of the three ARVs administrated by three different routes of administration to determine whether the selected doses result in plasma exposure equivalent to that observed in humans and (2) to determine the systemic levels of ARV drugs in our SIV-infected (SIV^+^) NHP model on long-term ART.

## 2. Materials and Methods

### 2.1. Animals

Cynomolgus macaques (*Macaca fascicularis*) were imported from Mauritius and housed at the IDMIT animal facility at the Commissariat à l’Energie Atomique et aux Energies Alternatives (CEA, Fontenay-aux-Roses, France). All NHP studies at the CEA are conducted in accordance with French National Regulations under the supervision of National Veterinary Inspectors (CEA accreditation number D92-032-02). The CEA complies with the Standards for Human Care and Use of Laboratory Animals of the Office for Laboratory Animal Welfare (OLAW, Bethesda, MD, USA) under OLAW Assurance number #A5826-01. All experimental procedures were conducted according to European Directive 2010/63 (Recommendation number 9). The study was approved by the ethics committee “Comité d’éthique en expérimentation animale dn°44” and the French Ministry of Education and Research under reference APAFIS#2453-2015102713323361.

Two groups of macaques were used in this study: 13 non-infected macaques and 12 macaques infected with the pathogenic SIVmac251 strain.

### 2.2. Antiretroviral Drug Formulations

The combined antiretroviral therapy (cART) consisted of a three-drug regimen comprised of TDF, FTC, and DTG. Three different routes of administration were compared: oral (PO), subcutaneous (SC), and intravenous (IV).

Treatment for the PO route was prepared by crushing commercially available 300 mg TDF and 50 mg DTG tablets and opening commercially available 200 mg FTC capsules and resuspending the powder in the flavoured oral suspending vehicle Ora-Blend (Perrigo Company plc, Allegan, MI, USA).

Solutions for the SC and IV routes were prepared by dissolving TDF, FTC, and DTG in sterile water using parenteral grade Kleptose HPB (Roquette Pharma, Lestrem, France), as previously described [21], to enhance the solubility and stability of the molecules.

### 2.3. Single-Dose Antiretroviral Drug Plasma Pharmacokinetic Study

To compare the pharmacokinetic parameters, 13 non-infected male cynomolgus macaques were assigned to three groups according to the route of administration. ARV doses were selected to approximate the human equivalent dose [22]. The oral formulation of TDF (44 mg/kg), FTC (50 mg/kg), and DTG (20 mg/kg) was administered once by gavage to two cynomolgus macaques. A single SC or IV injection of TDF (5.1 mg/kg), FTC (40.0 mg/kg), and DTG (2.5 mg/kg) was administered to seven and four cynomolgus macaques, respectively.

### 2.4. Repeated Antiretroviral Doses in Long-Term ART-Treated SIV-Infected NHP

To investigate the long-term administration of ARVs, 12 cynomolgus macaques were inoculated by the intravenous route with 1000 50% animal infectious doses (AID50) of cell-free SIVmac251 (kindly provided by A.M. Aubertin, Université Louis Pasteur, Strasbourg, France). The pathogenicity of this strain without antiretroviral treatment has already been shown, hence the absence of a control group [23].

The treatment was initiated 17 weeks post-infection, during the chronic phase of infection, for all NHPs [23]. Infected animals were assigned into two groups according to the route of ART administration at the time of initiation: six animals treated by the SC route and six animals by the PO route for the first eight weeks. For the PO route, ART was administered using an oral formulation of TDF (30 mg/kg), FTC (40 mg/kg), and DTG (20 mg/kg). For the SC route, the doses were 5.1/40.0/2.5 mg/kg for TDF/FTC/DTG, respectively. After eight weeks, all macaques were treated by the SC route for two years (Figure S1).

### 2.5. Blood Samples and Antiretroviral Drug Assays

For the single-dose pharmacokinetic study, blood samples were collected 0.25 (only for IV injection), 1, 2, 4, 6, and 24 h after ART administration.

For the long-term study, blood samples were collected just before ART administration and on days 1, 3, 8, 15, 21, 28, 43, and 58 and then every one or two months up to two years after treatment initiation. All animals were euthanised at the last sample time.

Blood samples were centrifuged at 4000× *g* for 10 min at +4 °C and the resulting plasma samples collected and stored at −80 °C until analysis. The concentrations of TNF, FTC, and DTG were measured simultaneously in plasma samples using an LC-MS/MS method, as previously described [24]. Briefly, 100 μL plasma was mixed with 300 μL acetonitrile containing deuterated internal standards. The samples were then vortexed and centrifuged and 50 μL of the supernatant mixed with 750 μL of an aqueous solution containing 0.1% formic acid. Finally, 20 μL was injected into the LC-MS/MS system (TSQ ALTIS, Thermo Fisher Scientific, Villebon- sur-Yvette, France).

### 2.6. Efficacy and Safety Assessments

Quantitative RT-PCR was performed on the plasma of ART-treated SIV^+^ NHPs to monitor plasma viral load (VL) using a SuperScript III Platinum One-Step qRT-PCR Kit (Thermo Fisher Scientific, Villebon-sur-Yvette, France) with a CFX96 Touch Real-Time PCR Detection System (Bio-Rad, Marnes-la-Coquette, France). Primary virological efficacy was assessed based on the change from pre-cART plasma VL to the VL at week 8 of cART, the last sampling before switching from the oral treatment to the SC route for the oral group. The secondary virological efficacy endpoint was the percentage of macaques with a plasma viral load <40 copies/mL at month 6 and at the end of the study.

The treatment-emergent adverse events were recorded to evaluate safety, both in the single-dose study and for the ART-treated SIV^+^ NHPs. In addition, given the known risk of a progressive decline in the estimated glomerular filtration rate (eGFR) linked to TDF-based regimens [25,26], renal safety was assessed by monitoring creatinine levels.

### 2.7. Pharmacokinetic Analysis

For the single-dose pharmacokinetic study, a non-compartmental analysis using Phoenix WinNonlin 8.1 (Certara, St. Louis, MO, USA) was performed to estimate the pharmacokinetic parameters. The observed parameters were the maximal concentration (C_max_), 24-h post-dose concentration (C_24h_), and time required to reach C_max_ (T_max_). The half-life (t_½_) was estimated from the log-linear terminal portion of the phase of decrease in concentration according to the formula t_½_ = 0.693/λz, where λz is the slope of the decrease in concentration. Areas under the curve (AUCs) were estimated using the linear up log down trapezoidal method until 24 h post dose (AUC_0→24h_).

For the repeated long-term pharmacokinetic study, the observed parameter was the trough concentration (C_t_).

Plasma C_24h_ or C_t_ were compared to wild-type HIV-1 90% inhibitory concentrations (IC_90_) for TNF (2.98 ng/mL) [27] and FTC (51 ng/mL) [28] and to the protein-adjusted IC_90_ (PA-IC_90_ = 64 ng/mL) for DTG [29].

### 2.8. Statistical Analysis

Intra-individual variability was calculated as the coefficient of variation of the C_t_ for each NHP from day 3 to the end of the study. For NHPs first treated via the oral route, intra-individual variability was calculated from day 3 to day 58 for the oral data and then from day 85 to the end of the study for the subcutaneous data. Inter-individual variability was calculated as the coefficient of variation of C_t_ at each time point from day 3 to the end of the study.

The data were analysed using GraphPad Prism (version 9.0.0 for Windows, GraphPad Software, San Diego, CA, USA). A Mann–Whitney test was performed to compare the inter- and intra-individual variability between the different routes of administration for the three ARVs. The threshold for statistical significance was set to *p* < 0.05. All numerical variables are expressed as medians and interquartile ranges (IQR) unless otherwise indicated.

## 3. Results

### 3.1. Single-Dose Antiretroviral Drug Plasma Pharmacokinetic Study

**Figure 1:**
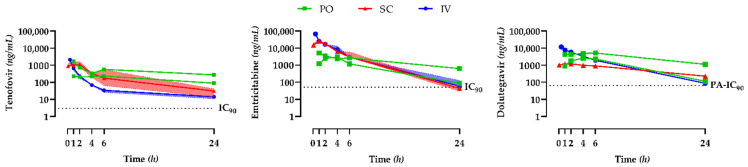
Tenofovir, emtricitabine, and dolutegravir total plasma pharmacokinetic profiles after a single dose. For the subcutaneous (SC, red) and intravenous (IV, blue) routes, the continuous lines and coloured areas represent the median and interquartile range, respectively. Individual data are represented for the oral (PO, green) route. Abbreviations: IC_90_: wild-type HIV-1 90% inhibitory concentration, PA-IC_90_: protein-adjusted wild-type HIV-1 90% inhibitory concentration.

Plots of the concentration against the time after IV, PO, and SC administration of TDF, FTC, and DTG are shown in Figure 1. Antiretroviral pharmacokinetic parameters are shown in Table 1.

The PO route showed large differences between the two macaques for the three drugs. The C_24h_ was above the IC_90_ for TNF and the PA-IC_90_ for DTG, regardless of the route of administration. For FTC, the C_24h_ was above the IC_90_, except for three macaques that received the ART by the SC route. TNF C_24h_ inter-individual variability was similar for the IV and SC routes (58%). DTG and FTC C_24h_ inter-individual variability was lower for the IV than the SC route: 108% vs. 59% for FTC and 26% vs. 13% for DTG.

For TNF and FTC, the t_½_ was similar for both the IV and SC routes. However, the t_½_ was higher for DTG given by the SC or PO route than by the IV route, suggesting a flip-flop phenomenon.

Except for DTG given by the SC route, the three drugs showed AUCs similar to or higher than those observed for humans. AUC inter-individual variability was lower for the IV than the SC route: 64% vs. 20% for TNF, 25% vs. 5% for FTC, and 19% vs. 6% for DTG. No adverse effects were recorded in this single-dose study.

### 3.2. Pharmacokinetics, Efficacy, and Safety in the Long-Term Treatment Model

**Figure 2:**
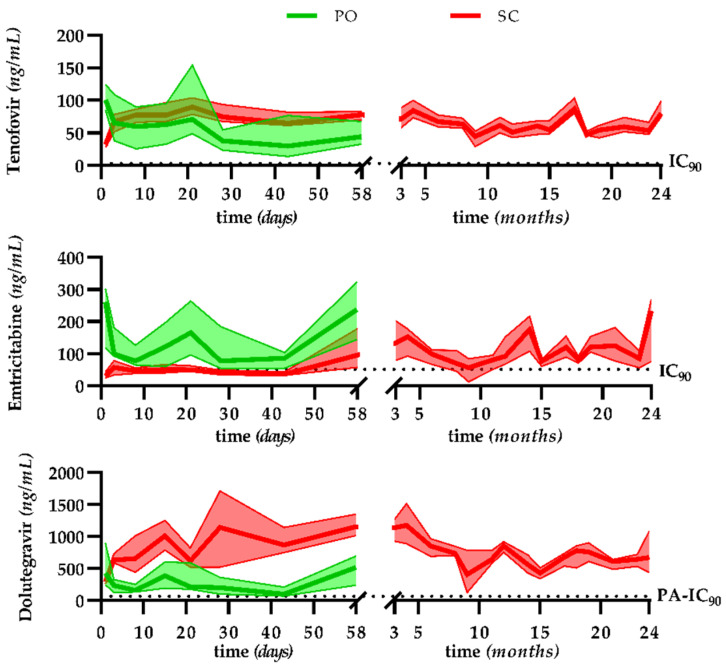
Tenofovir, emtricitabine, and dolutegravir trough plasma concentrations during long-term treatment of SIV^+^ macaques. The continuous lines and coloured areas represent the median and interquartile range, respectively. Oral (PO) route: green, subcutaneous (SC) route: red. Abbreviations: IC_90_: wild-type HIV-1 90% inhibitory concentration, PA-IC_90_: protein-adjusted wild-type HIV-1 90% inhibitory concentration.

The C_t_ for TNF, FTC, and DTG over two years of treatment is shown in Figure 2. Steady-state concentrations were reached for the three drugs for all animals from day 3. At steady state, all TNF and DTG C_t_ values (except one concentration of 49 ng/mL by the PO route on day 43) were above the IC_90_ or PA-IC_90_, respectively, regardless of the route of administration, PO or SC.

For FTC by the PO route, the C_t_ (except one concentration of 40 ng/mL on day 28 and one concentration of 28 ng/mL on day 43) was above the IC_90_. By the SC route, the C_t_ was close to the IC_90_ during the first 43 days and then above the IC_90_ throughout the rest of the treatment, except at month 9. At this time point, three macaques (25%) had exceptionally low C_t_ values (below 10 ng/mL).

**Figure 3:**
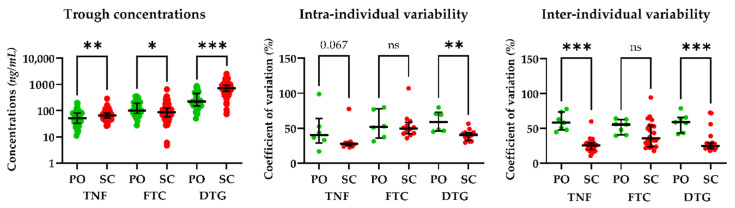
Steady-state trough concentrations depending on the route of administration and the intra- and inter-individual variability. The boxplot graphs represent trough concentrations (left), intra-individual variability (middle), and inter-individual variability (right); continuous lines represent the median and interquartile range values. Statistically significant differences are indicated as follows: * *p* < 0.05, ** *p* < 0.01, *** *p* < 0.001, ns: non-significant. Abbreviations: TNF: tenofovir, FTC: emtricitabine, DTG: dolutegravir, PO: oral route, SC: subcutaneous route.

The intra-individual and inter-individual variability of the C_t_ at steady state is shown in Figure 3. C_t_ values were significantly lower for the PO than the SC route for TNF (51 vs. 66 ng/mL, *p* = 0.007) and DTG (221 vs. 727 ng/mL, *p* < 0.001), but were significantly higher for FTC (86 vs. 100 ng/mL, *p* = 0.012).

Intra- and inter-individual variability were relatively low, with only one intra-individual coefficient of variation above 100%. There were no significant differences in the intra-individual variability between the SC and PO routes for TNF (*p* = 0.067) or FTC (*p* = 0.892) but it was significantly lower for the SC than the PO route for DTG (*p* = 0.003). The inter-individual variability was significantly lower for the SC than the PO route for TNF (*p* < 0.001) and DTG (*p* = 0.001) but was not significantly different for FTC (*p* = 0.079).

Plasma viral loads declined in all animals after the initiation of treatment. We observed a significant reduction in plasma viral load from baseline (before treatment initiation, median [IQR]: 4.3 [4.8] log10 copies/mL) to month 2. After two months under ART, all macaques had plasma viral loads <40 copies/mL, except one animal. This macaque belonged to the oral group and showed high viremia prior to treatment. Its plasma viral load became quickly undetectable after switching to the SC route. Antiviral drug pressure was then sustained without any viral rebound between month 6 and the end of the treatment phase for all NHPs, including the three macaques with FTC C_t_ values below the IC_90_ at month 9 (Table S1).

In terms of safety, few drug-related adverse events were reported during the entire follow-up period. Two adverse events were related to the treatment administration procedure (SC route for the majority of the study): induration or abscess and bleeding or hematomas. Diarrhoea was also observed, but even more rarely. None required treatment interruption, and all were self-limiting. After two years of treatment, changes in the level of the renal biomarker creatinine were small, with a median [IQR] increase of 2 [2] mg/L, i.e., 16.0% [23.8%]. The median [IQR] creatinine concentration was 13 [3] mg/L and was still within the usual biological reference values, except for two macaques with concentrations of 17 and 18 mg/L.

## 4. Discussion

In this study, we evaluated the plasma pharmacokinetics of TDF/FTC/DTG given simultaneously to healthy NHPs by three different routes of administration. We also reported the long-term plasma pharmacokinetics of these molecules in SIV^+^ NHPs. We thus demonstrated that it is possible to simultaneously and easily administer a three-antiretroviral-drug formulation at doses that enable a level of exposure close to that observed in humans over a two-year period with virological efficacy.

Developing and validating an NHP model for the long-term administration of three combined ARVs (two years) is of particular interest for further immunological, virological, and pharmacological studies.

Before validating our SIV^+^ long-term ART-treated NHP model, we determined the single-dose pharmacokinetics of the three selected drugs to validate the doses for the three routes of administration. These routes included that used clinically (PO), that used in preclinical studies in NHPs for long-term treatment (SC), and that used as the reference in pharmacokinetic studies (IV). They were tested to evaluate the difference between each main pharmacokinetic parameter. As ARVs may be considered as time-dependant anti-infective agents, the most important pharmacokinetic parameter is the C_24h_, which should be above the (PA-)IC_90_. The C_24h_ was above or close to the (PA-)IC_90_ for all three ARVs. For TNF and DTG, the C_24h_ was slightly lower than that observed in humans but was, nonetheless, above the respective IC_90_ or PA-IC_90_ [30,31]. For FTC, the C_24h_ was close to both that observed in humans and the IC_90_ [32]. For TNF and FTC, the high AUCs or C_max_ observed could be attributed to higher-weighted doses than used in humans [30,33]. However, no dose-effect toxicity was observed in our single-dose study. For DTG, the use of hydroxypropyl-beta-cyclodextrin and the SC route could be responsible for the observed flip-flop phenomenon, leading to a lower C_max_ and AUC and a higher t_½_ than expected and compared to those observed in humans [34,35,36,37].

The inter-individual variability for the AUC and C_24h_ was relatively low but higher for the oral than the SC and IV routes, the IV route showing the lowest inter-individual variability for the three ARVs studied. This can be explained by the different absorption mechanisms involved. Indeed, the oral route requires multiple mechanisms involved in drug absorption, whereas the SC route only requires that the drugs cross the capillary barrier without an intestinal or hepatic first-pass effect. For the IV route, the entire dose is immediately delivered to the systemic circulation without limiting factors. Overall, based on the C_24h_, the main pharmacokinetic parameter, all NHPs had concentrations within the expected therapeutic range for all three ARVs and the three routes of administration. Thus, the pharmaceutical formulations were validated. The C_24h_ for TDF and FTC was slightly higher for the oral route than that observed in humans and higher than that for the SC or IV routes in our study. Thus, the doses of these two ARVs were slightly reduced for the long-term study of the ART of SIV^+^ macaques.

We developed a model of long-term ART in macaques chronically infected with SIVmac251. At the time of ART initiation, we compared two routes of administration: the oral route, with the ARV administered in fruit, and the SC route. The IV route was not used for obvious reasons of ease of administration and the requirement of sedation. Compared to the administration by the oral route, the SC route has the advantage that the exact amount of drug intake is known and that no flavour adjustment is required. Flavour was a key point in the acceptance of oral treatment by the cynomolgus macaques. The oral suspending vehicle used had a sweet citrus-berry flavour, which appeared to be suitable.

Our data show rapid and sustained virological efficacy and good tolerance of the ARV treatments throughout the study. The slight increase in creatinine levels after two years under ART, although still within the usual biological reference values [38], is consistent with observed data in treatment-naive patients after 96 weeks [39,40]. This increase can be explained by two phenomena: the inhibition of the renal transporters SCL22A2 and SLC47A1 by DTG [41], both involved in the tubular secretion of creatinine [42,43], and the potential renal toxicity of tenofovir due to accumulation in the proximal tubular cells [25,26].

The novelty of our study consists of the pharmacological validation of the NHP model. In contrast to many studies that reported little or even no pharmacological data, we carried out regular long-term monitoring of the trough concentrations of the three ARVs used. Unlike the single-dose study, the C_t_ values of TNF and DTG were statistically higher following administration by the SC than the PO route. This can be explained by the use of hydroxypropyl-beta-cyclodextrin, an excipient for the SC formulation, which may be responsible for the flip-flop phenomenon and therefore produce a delayed-release form, increasing the drug accumulation ratio. However, despite these statistical differences between routes, all concentrations sampled 24 h after the previous administration were above the (PA-)IC_90_ throughout the study and consistent with clinical data. At month 9, three macaques had FTC C_t_ values below the IC_90_. However, a delay in the administration (median = 29 h 27 min, IQR = 1 min, after the previous one) and, therefore, in the residual sampling for this time point, could explain these low C_t_ values, also observed for TNF and DTG but with C_t_ values still above the (PA-)IC_90_. We observed no viral rebound around this sampling time for any of the three macaques. Therefore, these lower concentrations had no impact on the plasma viral load.

Only one previous study has reported long-term treatment and follow-up in NHPs [44]. The study was performed in rhesus macaques (*Macaca mulatta*) treated solely with TNF by the SC route and reported no trough concentrations, making a comparison with our results difficult. In addition, as for our single-dose study, the inter-individual variability was greater by the oral than the SC route. The potential impact of high doses on intestinal absorption, the hepatic first-pass effect, and the administration via the fruit with which the treatment is given may explain such differences.

Overall, our pharmacological data, associated with the robust viral suppression observed in the animals, allowed us to validate our long-term ART-treated SIV-infected macaque model. This model will be used for further pharmacokinetic/pharmacodynamic studies. We will characterize the diffusion of the three ARVs into different tissues to identify pharmacological sanctuaries. This model will also be a valuable tool to evaluate the impact of ARVs on viral reservoirs and on immunological parameters [45,46].

Our study had several limitations. First, the SC route is an injectable route. It is not the route used in humans for these ARVs and it may cause safety problems, such as injection-site pain, injection-site reactions, or a greater risk of infection. Nevertheless, only a few injection-site reactions and no infections arose during our two-year follow-up study. Moreover, being an injectable route, the SC route provides the advantage of lower intra- and inter-individual variability relative to the oral route and the precise dose administered is known. Second, the follow-up of the oral route arm was only carried out two months before the switch to the SC route. However, considering that the concentrations were close to those of the SC route arm and were virologically effective, the oral route also appears to be a potential route of administration for long-term follow-up.

Given that antiretroviral drugs are a long-term treatment modality for PLWHIV, our study, which highlights the pharmacokinetic and other advantages of oral and SC administration, makes an important contribution. This study provides the first description of long-term pharmacological exposure of a first-line worldwide treatment option administered simultaneously in an NHP model. Understanding the virus/drug interactions will be necessary to optimize newly developed ARV combinations. These data will have important implications towards an HIV cure.

## Figures and Tables

**Table 1:** Tenofovir/emtricitabine/dolutegravir single-dose pharmacokinetic parameters.

	C_max_ (ng/mL)	T_max_ (h)	C_24h_ (ng/mL)	t_½_ (h)	AUC_0→24h_ (ng·h/mL)
**Tenofovir**					
PO (n = 2)	1668 231	1.0 6.0	272 88	31.0 14.2	11,115 3857
SC (n = 7)	1103 (954–1654)	1.0 (1.0–2.0)	32 (16–50)	4.7 (1.2–6.1)	4391 (2326–8598)
IV (n = 4)	2096 (1583–2352)	0.25 (0.25–0.25)	21 (12–38)	5.1 (1.4–14.1)	1983 (1825–2570)
**Emtricitabine**					
PO (n = 2)	5062 2957	1.0 4.0	632 87	9.6 4.8	42,768 19,280
SC (n = 7)	25,882 (19,607–28,634)	1.0 (1.0–1.0)	44 (29–61)	3.1 (2.6–3.2)	79,578 (76,106–96,001)
IV (n = 4)	67,732 (62,920–70,569)	0.25 (0.25–0.25)	58 (54–127)	3.0 (2.8–3.2)	111,530 (106,512–116,484)
**Dolutegravir**					
PO (n = 2)	5105 2550	6.0 4.0	1109 113	8.2 4.1	72,426 23,956
SC (n = 7)	1229 (1082–1467)	1.0 (1.0–2.0)	224 (180–281)	9.2 (8.8–9.7)	15,352 (11,401–16,540)
IV (n = 4)	11,643 (10,995–12,583)	0.25 (0.25–0.25)	86 (82–102)	3.8 (3.7–4.2)	39,601 (37,865–42,241)

Variables are expressed as medians and interquartile ranges (IQR) when *n* ≥ 3. For the oral (PO) route, individual values are expressed. Abbreviations: PO: oral route, SC: subcutaneous route, IV: intravenous route, C_max_: maximal concentration, C_max_ values for intravenous administration were observations at 0.25 h (as first point of blood sampling), T_max_: time to reach C_max_, C_24h_: 24-h post-dose concentration, t_½_: half-life, AUC_0→24h_: area under the curve until 24 h post dose.

## Data Availability

The data presented in this study are available on request from the corresponding author. The data are not publicly available due to privacy.

## Supplementary Materials

The following supporting information can be downloaded at: https://www.mdpi.com/article/10.3390/pharmaceutics14112282/s1, Figure S1: Repeated-dose study design, Table S1: Virological follow-up.
