# Supplementary material for: Pharmacological Validation of Long-Term Treatment with Antiretroviral Drugs in a Model of SIV-Infected Non-Human Primates
Source: Pharmaceutics. 2022 Oct 25;14(11):2282. doi: 10.3390/pharmaceutics14112282 (PMC9696548; doi:10.3390/pharmaceutics14112282)
Supplement: Supplementary file 1 [file pharmaceutics-14-02282-s001.zip › pharmaceutics-1959858-supplementary.pdf]

pharmaceutics

Pharmacological validation of long-term treatment with antiretroviral drugs in a model of SIV-infected non-human primates

Thibaut Gelé, Hélène Gouget, Nathalie Dereuddre-Bosquet, Valérie Furlan, Roger Le Grand, Olivier Lambotte, Delphine Desjardins and Aurélie Barrail-Tran

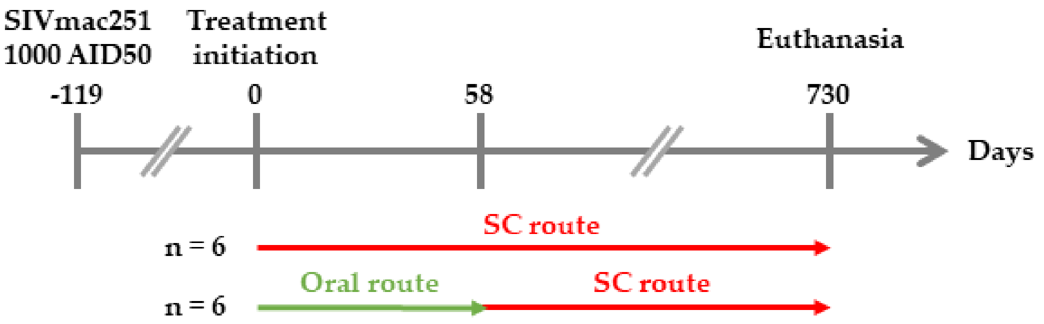

Doses (mg/kg/day)	TDF	FTC	DTG
SC route	5.1	40	2.5
Oral route	30	40	20

Figure S1. Repeated-dose study design. Abbreviations: AID50: 50% animal infectious doses, SC: subcutaneaous, TDF: tenofovir disproxil fumarate, FTC: emtricitabine, DTG: dolutegravir.

Table S1. Virological follow-up.

	Plasma viral load (log_{10} copies/mL)	Percentage of NHPs with undetectable plasma viral load (threshold = 40 copies/mL = 1.6 log_{10} copies/mL)			
	Baseline	Month 2	Month 6	Month 12	End of treatment
PO (n = 6)	4.3 (4.6)	83 %	/	/	/
SC (n = 6)	4.4 (4.9)	100 %	/	/	/
Total (n = 12)	4.3 (4.8)	92 %	100 %	100 %	100 %

Variables are expressed as medians and interquartile ranges (IQR). Abbreviations: PO: oral route, SC: subcutaneous route, IV: intravenous route, NHP: non-human primate.
